# Evaluating the impact of the universal infant free school meal policy on the ultra-processed food content of children’s lunches in England and Scotland: a natural experiment

**DOI:** 10.1186/s12966-024-01656-w

**Published:** 2024-11-01

**Authors:** Jennie C. Parnham, Kiara Chang, Fernanda Rauber, Renata B. Levy, Anthony A. Laverty, Jonathan Pearson-Stuttard, Martin White, Stephanie von Hinke, Christopher Millett, Eszter P. Vamos

**Affiliations:** 1 Department of Primary Care & Public Health, Public Health Policy Evaluation Unit, School of Public Health, Imperial College London, 90 Wood Lane, London, W12 0BZ UK; 2 Center for Epidemiological Research in Nutrition and Health, University of São Paulo, São Paulo, 01246-904 Brazil; 3 Department of Preventive Medicine, School of Medicine, University of São Paulo, São Paulo, 01246-903 Brazil; 4 Department of Epidemiology and Biostatistics, School of Public Health, Imperial College London, London, SW7 2AZ UK; 5 Northumbria Healthcare NHS Foundation Trust, Newcastle upon-Tyne, NE27 0QJ UK; 6 Health Analytics, Lane Clark & Peacock LLP, London, W1U 1DQ UK; 7 MRC Epidemiology Unit, University of Cambridge, Cambridge, CB2 0QQ UK; 8 School of Economics, University of Bristol, Priory Road Complex, Bristol, BS8 1TU UK; 9 Public Health Research Centre & Comprehensive Health Research Center (CHRC), National School of Public Health, NOVA University of Lisbon, Lisbon, 1600-1500 Portugal

**Keywords:** School food, Universal free school meals, Policy evaluation, Children, Ultra-processed food

## Abstract

**Background:**

The Universal Infant Free School Meal (UIFSM) policy was introduced in 2014/15 in England and Scotland for schoolchildren aged 4–7 years, leading to an increase in school meal uptake. UK school meals are known to be healthier and less industrially processed than food brought from home (packed lunches). However, the impact of the UIFSM policy on the quantity of ultra-processed food (UPF) consumed at school during lunchtime is unknown. This study aimed to evaluate the impact of the UIFSM policy on lunchtime intakes of UPF in English and Scottish schoolchildren.

**Methods:**

Data from the UK National Diet and Nutrition Survey (2008–2019) were used to conduct a difference-in-difference (DID) natural experiment. Outcomes included school meal uptake and the average intake of UPF (% of total lunch in grams (%g) and % total lunch in Kcal (%Kcal)) during school lunchtime. The change in the outcomes before and after the introduction of UIFSM (September 2014 in England, January 2015 in Scotland) in the intervention group (4–7 years, *n =* 835) was compared to the change in an unexposed control group (8–11 years, *n =* 783), using linear regression. Inverse probability weights were used to balance characteristics between intervention and control groups.

**Results:**

Before UIFSM, school meal uptake and consumption of UPFs were similar in the intervention and control groups. The DID model showed that after UIFSM, school meal uptake rose by 25%-points (pp) (95% CI 14.2, 35.9) and consumption of UPFs (%g) decreased by 6.8pp (95% CI -12.5,-1.0). Analyses indicated this was driven by increases in minimally processed dairy and eggs, and starchy foods, and decreases in ultra-processed salty snacks, bread and drinks. The differences were larger in the lowest-income children (-19.3 UPF(%g); 95% CI -30.4,-8.2) compared to middle- and high-income children. Analyses using UPF %Kcal had similar conclusions.

**Conclusions:**

This study builds on previous evidence suggesting that UIFSM had a positive impact on dietary patterns, showing that it reduced consumption of UPFs at school lunchtime, with the greatest impact for children from the lowest-income households. Universal free school meals could be an important policy for long term equitable improvements in children’s diet.

**Supplementary Information:**

The online version contains supplementary material available at 10.1186/s12966-024-01656-w.

## Background

The consumption of ultra-processed food (UPF) in British children is concerning: over 65% of UK children’s diet comes from UPFs [1], which is higher than both British adults [2] and intakes in other European countries [3, 4]. UPFs are foods which have undergone extensive industrial processing, they are designed to be attractive, convenient, cheap, and are highly marketed [4, 5]. Typically, UPFs are foods with poor nutritional profiles, have artificial, non-nutritive ingredients and little resemblance to whole foods. Evidence now indicates that UPFs are associated with negative consequences for both child [6] and adult health [7]. Long-term health impacts of UPFs include increased weight gain [8], type 2 diabetes [9], cardiovascular disease [4], cancer [10], and premature mortality [11]. Furthermore, UPFs are consumed disproportionately by children from lower socio-economic backgrounds [2, 8, 12]. Therefore, it is vital to identify ways to lower UPF intake in children’s diets.

Schools are a critical setting to influence children’s diet as children consume one third of their weekday diet at school [13]. However, the dietary quality of foods eaten at schools has been found to vary according to whether a child eats a school meal or food brought from home, referred to as a packed lunch [12, 14, 15]. Packed lunches in the UK, on average, have a less favourable nutrient and food content and are a higher source of UPF than school meals [12, 15]. Foods manufactured specifically for children are heavily marketed by UPF companies [16] and parents report being influenced by their children’s desire for these products [17]. Conversely, school meals are on average lower in UPF and are likely less impacted by marketing influences. Therefore, action to increase school meal uptake could be one method to reduce UPF intake in children.

In September 2014 in England and January 2015 in Scotland, the Universal Infant Free School Meal (UIFSM) policy was introduced for children in the first three years of school (Key Stage one (KS1), roughly 4–7 years) [18]. However, a means-tested system remained for older children (roughly over 8 years) whereby only children whose family received certain social security benefits were eligible for a free school meal. The UIFSM policy increased school meal uptake by around 50%-points (pp) in those not previously eligible [19] and has been shown to have a positive impact on the nutritional content of children’s lunches and their bodyweight [19, 20]. The policy has since been expanded in some areas of the UK (Scotland, Wales and London) to include all primary school children (ages 4–11 years) [21]. However, it is not known how the policy impacted consumption of UPFs. We hypothesised that the introduction of the UIFSM policy was associated with a reduced UPF intake in schoolchildren. Furthermore, as UPF intake is socially patterned [12], we hypothesised that the UIFSM policy may have a greater impact on UPF intake in children from lower income households compared to children from higher income households.

This study aims to assess the impact of the introduction of the UIFSM policy in 2014/15 on the quantity and variety of UPF consumed at lunchtime among schoolchildren in England and Scotland.

## Methods

### Study design

This study used a difference-in-differences (DID) study design to estimate the impact of the UIFSM policy on children’s ultra-processed food intake during school lunchtime. This was done by comparing average changes in lunchtime intakes consumed pre-UIFSM and post-UIFSM (September 2014 for England, January 2015 for Scotland) between intervention (KS1 children, roughly ages 4–7 years) and control groups (children in the subsequent four years of school, termed Key Stage two (KS2), roughly 8–11 years). KS2 schoolchildren were the most appropriate control available as they were not eligible for UIFSM but were in the same primary school environment. In other words, the study design assesses the impact of school meals being universally free to KS1 schoolchildren compared to the previous means-tested system. In the DID design, we assume that any change in dietary intake in the intervention group would have been the same in the control group in absence of the UIFSM policy (i.e. parallel trends assumption holds). Furthermore, this study estimates the intention-to-treat (ITT) effect. While children may have been exposed to the UIFSM policy, they could still choose not to take their free school meal and bring a packed lunch instead.

### Data sources

This study used data from the National Diet and Nutrition Survey (NDNS, years 2008–2019) [22], which is a repeated annual cross-sectional survey that collects a representative record of individuals’ dietary intake in the UK (aged over 1.5 years). Clustered random sampling was used to invite households from a national list of postcodes; further details are described elsewhere [23]. A consecutive three or four-day paper diary was used to record dietary data, which recorded the location, time and quantity of all food and drink consumed. Diaries were filled out by a guardian due to participant’s age (all less than 12 years). A carer pack was issued, which included a separate recording sheet and an information leaflet, so that a carer (e.g. a teacher) could complete sections of the diet diary for the child when they were away from home [24]. Participants were asked to fill in details to help the coding of foods, including brand names (where applicable), food label information for ready meals and unusual foods and whether the item was homemade (including cooking method). The DINO system (Diet In Nutrients Out) was used to convert food and drink data to nutrient data using Public Health England’s NDNS Nutrient Databank, with full details on the coding of dietary data published elsewhere [24]. A trained interviewer collected additional variables, including sociodemographic information.

### Study participants

The study population included all KS1 and KS2 schoolchildren (4–11 years) in England or Scotland. From the initial sample (*n* = 2,194), participants were excluded if their school did not provide food (4%, *n* = 78), they did not record eating a lunch at school in their dietary diary (i.e. due to school holidays, 22%, *n* = 480) or they did not report a school meal type (1%, *n* = 18), leaving an analytic sample of 1,618 participants (intervention *n =* 835; control *n =* 783).

### Exposure variables

To define the exposure variable, participants were categorised into two time-periods: pre-UIFSM and post-UIFSM. If participants in England recorded their dietary data before September 2014 (January 2015 for participants in Scotland), they were classified as pre-UIFSM (2008–2014/15). Post-UIFSM was classified as dietary data recorded after these dates, dependent on the country (2014/15-2019). The sample was divided into two time-periods due to the relatively small annual samples in the intervention and control groups, which precluded a time series analysis. Binary variables for time-period (0 = pre-UIFSM,1 = post-UIFSM) and intervention group (0 = control, 1 = intervention) were created.

### Outcome variables

The lunchtime variables only included food and drink items recorded between 11:30 − 14:00pm on a weekday at a school premises. Lunchtime intakes were averaged where multiple days were recorded (1 school day [*n* = 207, 13%], 2 school days [*n* = 676, 42%], 3 school days [*n* = 411, 25%], 4 school days [*n* = 324, 20%])

The level of industrial processing of foods was assessed using the Nova classification system [25]. This categorises foods into four groups by the extent and purpose of processing:

1. Non or minimally processed foods (MPF): Foods which have undergone no or minimal processing from their original or whole state. Processing is to make the food edible or safe (including boiling, frying, pasteurising).
2. Culinary ingredients: Includes foods which are used in food preparation. They may be substances extracted from nature (salt) or be substances derived from group 1 foods (butter, olive oil).
3. Processed foods: Foods which have undergone processing using traditional methods (e.g. fermenting, salting) that combines MPF and culinary ingredients (e.g. salt, vinegar, sugar and fat).
4. Ultra-processed foods (UPF): Foods which have undergone extensive industrial processing and contain little or no whole foods. They may contain ingredients extracted from whole foods and artificial, non-nutritive ingredients.

The categorisation of NDNS items according to the Nova classification is detailed elsewhere [2]. In brief, information given, including the cooking method, brand name, food name and recipe description, were used to categorise the food according to processing level. The primary outcome measures were the contribution of MPF and UPF to the total food weight (%g) and total energy (%kcal) consumed at school lunchtime.

The secondary outcome measures were the Nova subgroups. The MPF subgroups included minimally processed drinks, fruit and vegetables, dairy products, starchy products, minimally processed meat, and fish products and the UPF subgroups included ultra-processed drinks, ultra-processed bread, salty snacks, sweet foods [including sweet snacks and puddings], ready-to-eat foods [including fast-foods and condiments], ultra-processed dairy, meat and fish, ultra-processed vegetables [baked beans, i.e. beans in an ultra-processed tomato sauce]. See Supplementary Table 8 for full descriptions. Consumption of ‘Culinary ingredients’ (Nova group 2) and ‘Processed foods’ (Nova group 3) was too small to describe subgroups, therefore these groups were shown in aggregate. For the regression analysis, Nova subgroups were dichotomised into consuming none (0 g/lunch) or some (> 0 g/lunch) due to the variables being skewed toward zero.

The contribution of MPF and UPF to the total daily energy (%kcal day) and weight consumed (%g day) were also calculated. This was to test if the UIFSM policy impacted total dietary intakes across the school day in addition to during lunchtime only.

### Covariates

Covariates included age (years); sex, ethnicity (White or Ethnic Minority groups); equivalized household income (tertiles); Index of Multiple Deprivation (IMD) (quintiles); country (England or Scotland) and total lunchtime intake (g/lunch or Kcal/lunch, dependent on the outcome variable). Household income was adjusted by the McClements Equivalence Scales [23, 26] by NDNS, to account for a household’s size and composition. IMD is an area-based composite measure of relative deprivation in the UK [27]. Study covariates were complete except for household income (*n* = 171 missing, 11%), ethnicity (*n* = 1 missing) and IMD (*n* = 1 missing). Missing variables were imputed using the Classification and Regression Trees (CART) method using 10 iterations [28].

### Statistical analysis

Sample characteristics were compared in each intervention group and time-period using a Wald test, adjusted by survey weights. The average intake of MPF and UPF including the contribution of Nova subgroups, adjusted by survey weights, was presented graphically for the two groups and time-periods. Survey-weighted t-tests were used to determine if the difference in the average level of outcome for each group before and after the policy were statistically significant.

Linear regression models were used to assess the UIFSM impact on lunchtime MPF and UPF consumption, and total daily consumption of UPF and MPF. Linear probability models were used to assess changes in the probability taking a school meal and of consuming a Nova subgroup at lunchtime. The models included the indicators for time-period, intervention group and an interaction between these terms. The latter was the DID estimator that compares average changes in the outcome between the pre- and post-UIFSM period in the intervention and control groups to estimate the impact of the policy. The model uses the control group as the best estimate of counterfactual outcome for the intervention group, in the absence of the policy.

Three regression models are presented. Model 1 is an unadjusted DID model, Model 2 is adjusted for the sociodemographic covariates and Model 3 is additionally adjusted for total lunchtime intake (g/lunch or Kcal/lunch, dependent on the outcome variable). The analyses were further stratified by income tertiles (low, medium, and high) to investigate if lower-income children were differentially impacted compared to higher-income children.

Inverse probability weights (IPW) were computed to ensure covariates were balanced across each group [29]. The IPW were calculated as the inverse of the predicted probability of being in the pre-intervention group across the four groups (pre-intervention, pre-control, post-intervention, post-control) using a multinomial regression model including sex, ethnicity, country, household income, IMD, socioeconomic status, and the provided survey weight variable. All models were weighted using the IPW.

Sensitivity analyses were conducted to assess if the findings were robust to different sources of bias. Dietary misreporting was identified by comparing participant’s estimated energy requirements to their reported energy intake, using the Goldberg method, adapted for children [30, 31]. In the sample, 4% (*n =* 66) were estimated to be unreliable energy reporters. The analyses were repeated excluding these participants. Furthermore, participants with only one day of dietary data (*n* = 207) were excluded and the analysis was repeated.

## Results

### Sample characteristics and school meal uptake

There were 1,618 participants included in the study, of whom 966 (60%) were in the pre-UIFSM period and 835 (52%) were in the intervention group (Table 1). Sociodemographic characteristics were balanced across the exposure groups. Application of IPW further reduced mean differences across all sample characteristics between intervention groups and periods.

**Table 1:** Characteristics of schoolchildren (*n =* 1,618) in England and Scotland before and after the UIFSM policy

		Pre-UIFSM (2008–2014)		Post-UIFSM (2014–2019)^1^		
Characteristic		Intervention group (*n* = 495, 51.2%)	Control group (*n* = 471, 48.8%)	Intervention group (*n* = 340, 52.1%)	Control group (*n* = 312, 47.9%)	*P*-value^2^
**Sex**	n (%)					0.587
Male		255 (53%)	257 (55%)	168 (50%)	167 (52%)	
Female		240 (47%)	214 (45%)	172 (50%)	145 (48%)	
**Ethnicity**	n (%)					0.319
White		421 (83%)	407 (83%)	274 (78%)	251 (78%)	
Ethnic minorities		74 (17%)	64 (17%)	66 (22%)	61 (22%)	
**Household Income (tertiles)**	n (%)					0.627
Lowest		154 (34%)	145 (32%)	90 (31%)	105 (37%)	
Middle		185 (38%)	193 (38%)	130 (35%)	110 (33%)	
Highest		156 (28%)	133 (29%)	120 (34%)	97 (29%)	
**IMD (quintiles)**	n (%)					0.882
Most deprived		106 (23%)	103 (23%)	82 (23%)	69 (23%)	
2		93 (19%)	87 (17%)	72 (22%)	59 (18%)	
3		100 (19%)	95 (19%)	56 (17%)	64 (20%)	
4		88 (19%)	80 (18%)	64 (20%)	48 (16%)	
Least deprived		108 (21%)	106 (24%)	66 (19%)	72 (23%)	
**Country**	n (%)					0.452
England		374 (91%)	329 (90%)	312 (93%)	280 (90%)	
Scotland		121 (9.5%)	142 (10%)	28 (7.3%)	32 (9.8%)	
**School lunch type**	n (%)					< 0.001
School meal		220 (46%)	193 (39%)	270 (78%)	156 (50%)	
Packed lunch		275 (54%)	278 (61%)	70 (22%)	156 (50%)	

^1^ Threshold is September 2014 for English participants and January 2015 for Scottish participants

^2^ Wald test for difference across four groups (adjusted for complex survey sample)

Note: UIFSM -Universal Infant Free School Meal; Intervention – Infants (4–7 years); Control – juniors (8–11 years); IMD – Index of Multiple Deprivation

### UIFSM policy impact on uptake of school meals

In the pre-UIFSM period, take-up of school meals was similar between intervention and control groups (44.4% vs. 41.6%, respectively, see Supplementary Table 1). However, in the post-UIFSM period the take-up of school meals was significantly higher in the intervention group (76.8%) than the control (49.3%). In a fully adjusted DID model, UIFSM was shown to increase school meal uptake by 25%-points (pp) (95% CI 14.2, 35.9). The estimates between the unadjusted and adjusted models were similar.

### Before-after differences in lunchtime outcomes

Before UIFSM, lunchtime intakes of the intervention and control groups were similar (Supplementary Table 2). For example, the intervention group consumed 51.6% (SD 26.2) of their lunch as UPF (% g) and the control group consumed 50.9% (SD 27.1) of their lunch as UPF (% g). However after UIFSM, UPF intakes (% g) were 12%-points (pp) (95% CI -16.0,-7.3) less in the intervention group compared to the pre-intervention period, with no evidence of a change in the control group. Results were similar for UPF measured as a percentage of energy (% kcal).

**Fig. 1:**
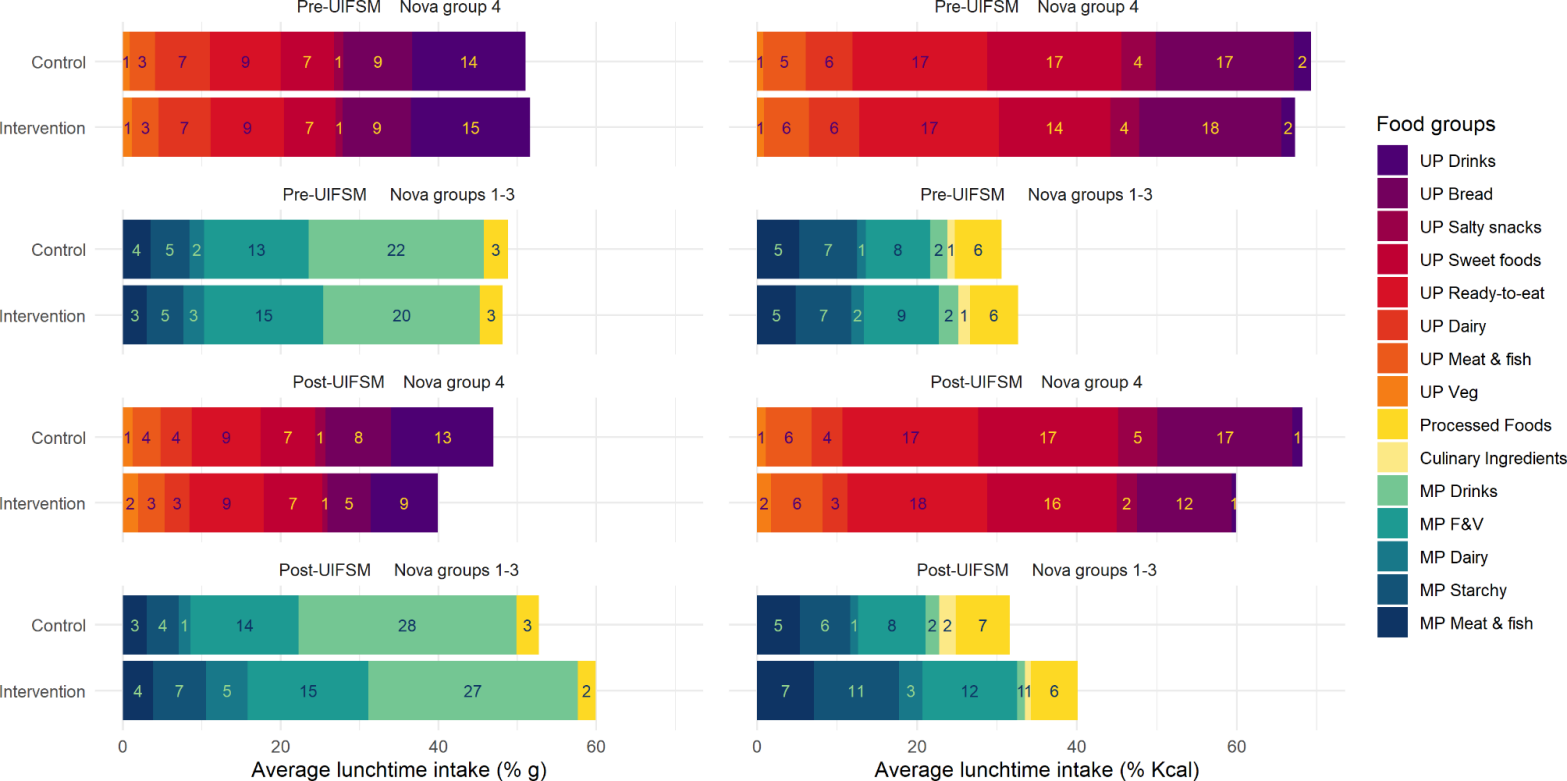
The composition of lunchtime intakes of schoolchildren (*n* = 1,618) in England and Scotland before and after the UIFSM policy by the level of industrial food processing Note: Intervention = 4–7-year-olds; Control = 8–11-year-olds; Pre- UIFSM = Before introduction of the Universal Infant Free School Meal policy (2008–2014/15); Post- UIFSM = After introduction of the Universal Infant Free School Meal policy (2014/15-2019); Nova group 4 = Ultra-processed foods; Nova groups 1–3 = Minimally professed foods, Culinary ingredients, and Processed foods; MP = Minimally processed; UP = Ultra-processed; % grams = Percent of total lunchtime grams; % kcal = Percent of total lunchtime calories

When the contribution by food groups to lunchtime intake was described, a similar trend was observed (Fig. 1). The pre-intervention lunch showed similar food group intakes between the intervention and control groups; UPF drinks (15.1%g intervention vs. 14.3%g control), UPF bread (8.6%g vs. 8.7%g) and UP ready-to-eat foods (9.3%g vs. 8.9%g) were the biggest source of UPFs. However, after UIFSM, the intervention group had a higher intake of minimally processed dairy (5.3%g intervention vs. 1.7%g control), and starchy foods (6.7%g vs. 4.1%g) and a lower intake of UP drinks (8.6%g vs. 13.0%g) and bread (5.4 g vs. 8.3%g) than the control group. These patterns were similar when the contribution of food groups to energy intake was analysed.

### UIFSM policy impact on the quantity of industrially processed food consumed during school lunchtime

**Fig. 2:**
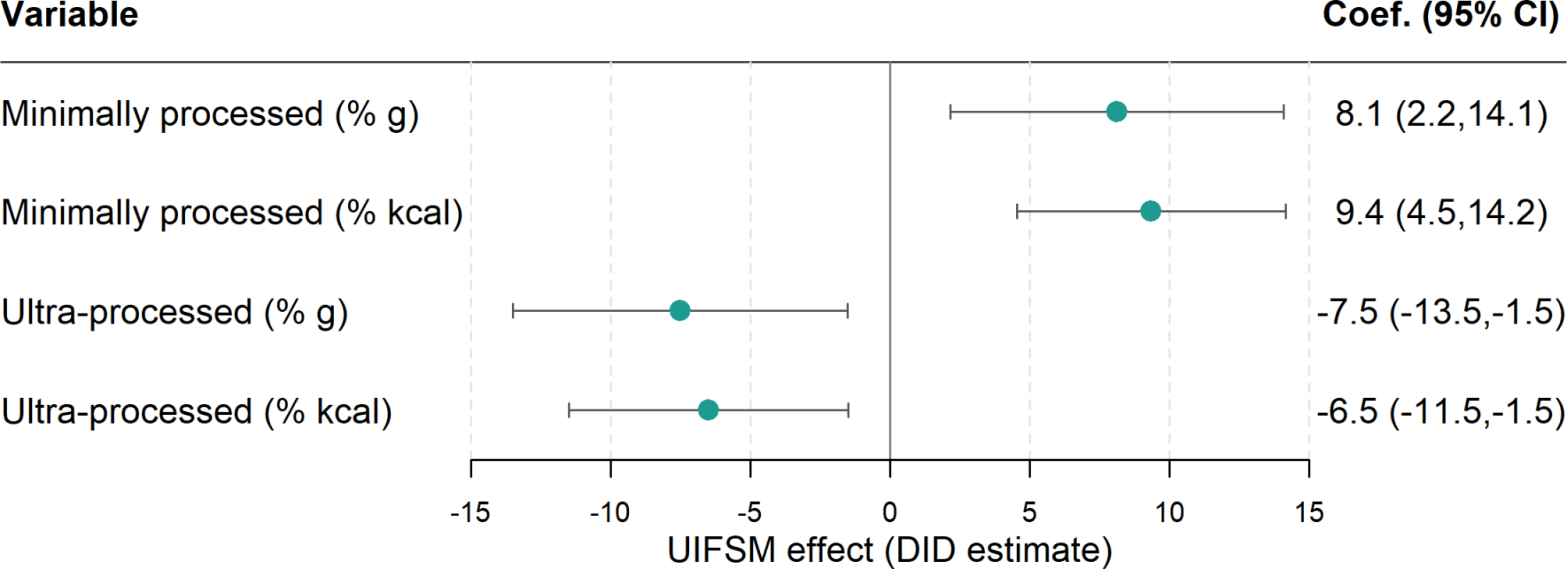
Estimates of the effect of the UIFSM policy on the proportion of minimally processed foods and ultra-processed foods of foods consumed at lunchtime in a sample of schoolchildren (*n* = 1,618) Note: Linear regression adjusted for age, sex, ethnicity, country, household income and IMD (Model 2). CI - confidence interval; %g – Percent of total lunchtime grams; % kcal – Percent of total lunchtime calories. Pre/Post average values are presented in Supplementary Table 2

The policy impact of UIFSM on the quantity of UPF and MPF consumed during school lunchtime was estimated using a DID model (Fig. 2). After adjustment for socio-demographic covariates (Model 2, Supplementary Table 2), UIFSM was associated with 7.5 pp (95% CI -13.5,-1.5) less UPF by weight (%g) and 6.5 pp (95% CI -11.5, -1.5) less UPF consumption by energy (% Kcal). This corresponded to changes in MPF consumption, with an 8.1 pp (95% CI 2.2, 14.1) increase by weight (%g) and a 9.4 pp (95% CI 4.5, 14.2) increase by energy (% Kcal). Additional adjustment for total lunchtime dietary intake (grams or energy, respectively) to further account for differences in food intake between children were not found to statistically impact the estimates.

### UIFSM policy impact on the type of minimally and ultra-processed food groups consumed during school lunchtime

**Fig. 3:**
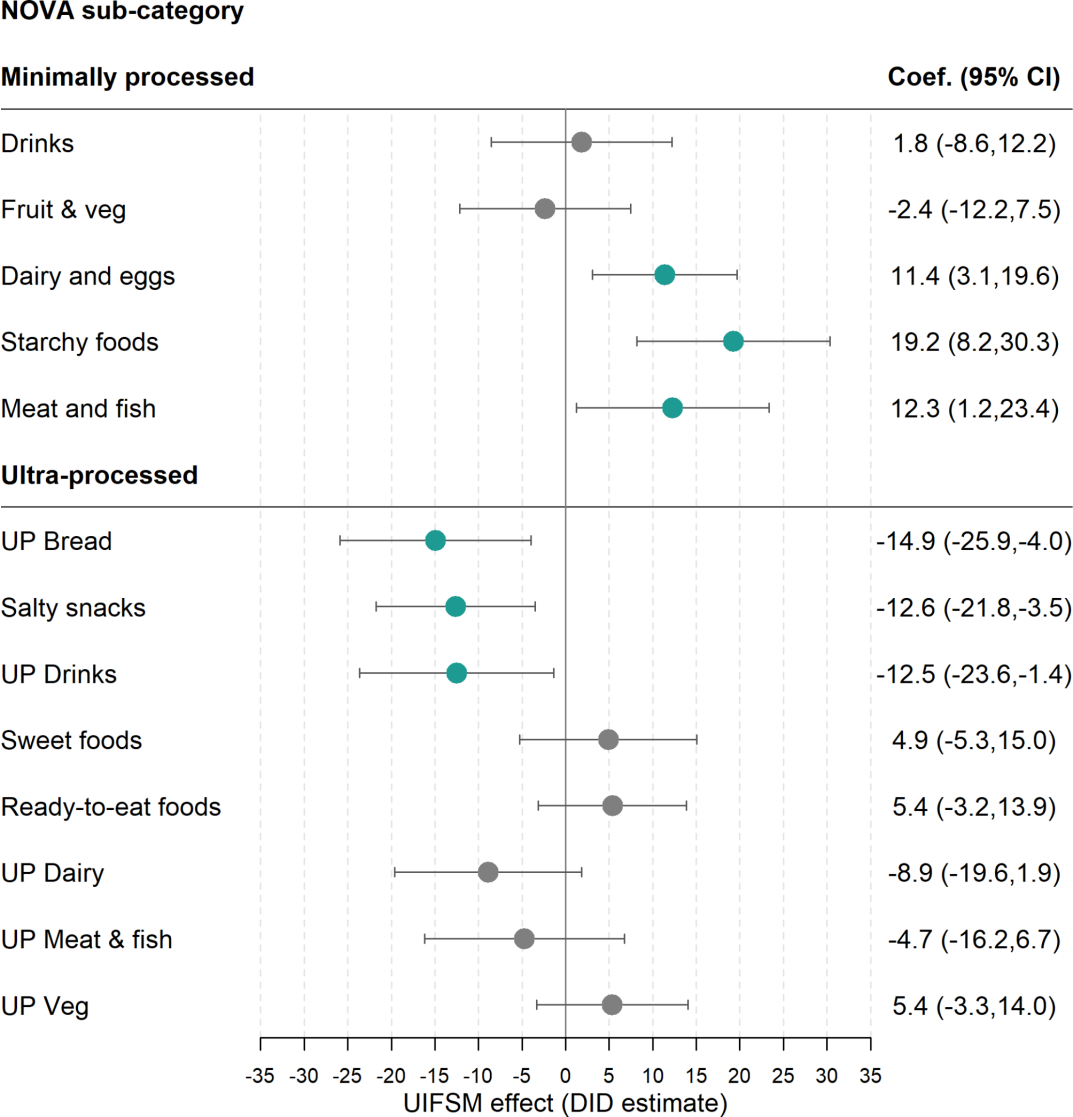
Estimates of the effect of the UIFSM policy on the proportion of children consuming minimally and ultra-processed food groups at lunchtime in a sample of schoolchildren (*n* = 1,618) Note: Linear probability regression adjusted for age, sex, ethnicity, country, household income and IMD Coefficients refer to the percentage-point change in prevalence of children consuming a food group. Pre/Post average consumption of minimally and ultra-processed food groups are presented in Supplementary Table 3

The impact of the UIFSM policy on the likelihood of consuming different minimally and ultra-processed food groups at school lunchtime was also estimated using the DID model (Fig. 3, Supplementary Table 3). This model compares the proportion of children of eating some (> 0 g) of the food group to the proportion eating none (0 g). After full adjustment of covariates, UIFSM increased the proportion of children consuming any amount of minimally processed dairy and eggs (11.4 pp, 95% CI 3.1,19.6), starchy foods (19.2 pp, 95% CI 8.2,30.3) and meat and fish (12.3 pp, 95% CI 1.2,23.4). There was also a reduction in the likelihood that children consumed ultra-processed bread (-14.9 pp, 95% CI-25.9, -4.0), salty snacks (-12.6pp, 95% CI -21.8, -3.5) and ultra-processed drinks (-12.5pp, 95% CI -23.6,-1.4). There was no change in minimally processed fruit and vegetables or ultra-processed food groups such as sweet foods and ready-to-eat foods, which included fast foods.

### Differences of UIFSM policy impact by income group

**Fig. 4:**
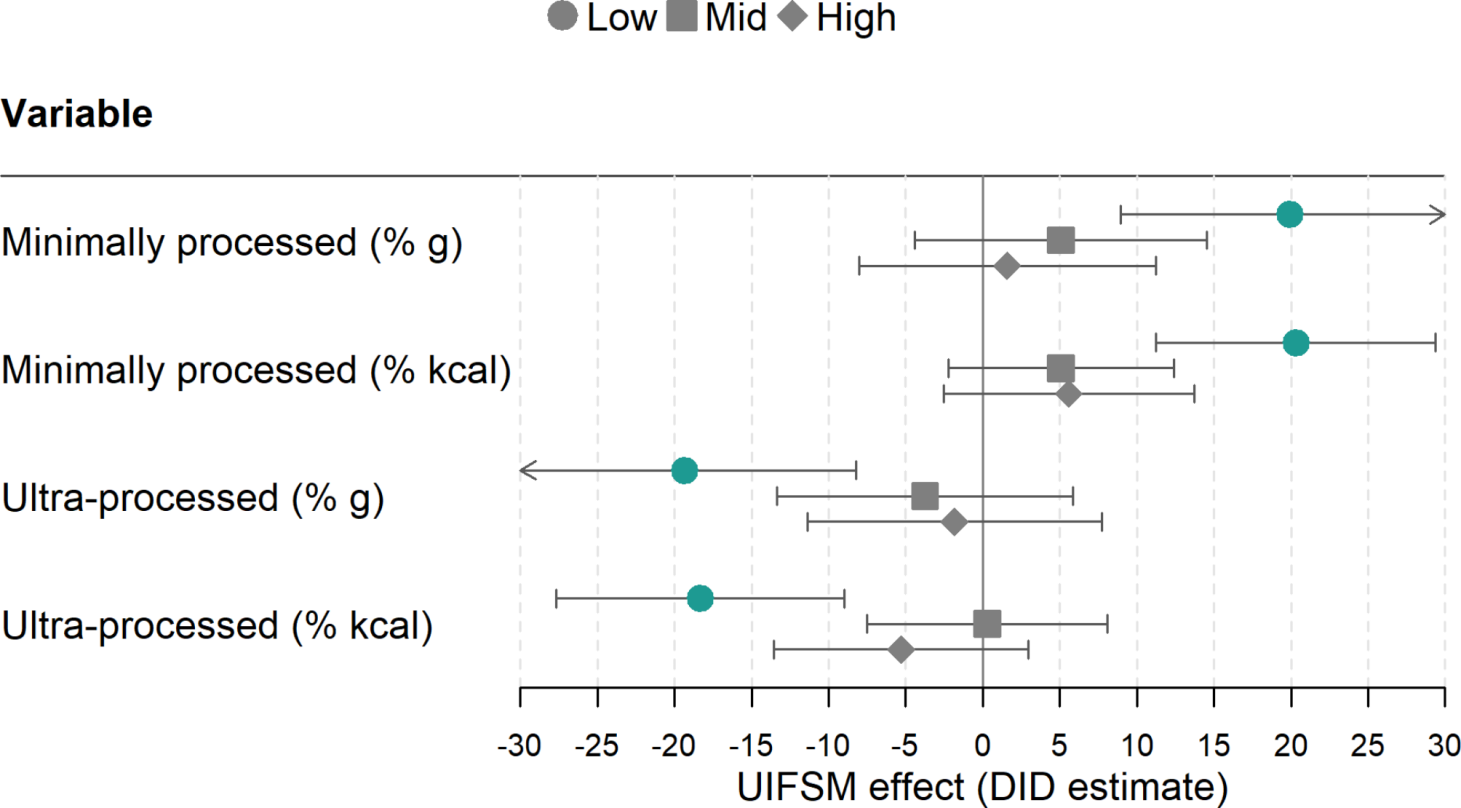
Estimates of the effect of the UIFSM policy on the proportion of minimally processed and ultra-processed foods consumed at lunchtime in a sample of schoolchildren (*n* = 1,618) and stratified by household income (tertiles) Note: Linear regression adjusted for age, sex, ethnicity, country, household income and IMD. %g – Percent of total lunchtime grams; % kcal – Percent of total lunchtime calories; The pre-post UIFSM average consumption of minimally and ultra-processed food for each income tertile are presented in Supplementary Table 4

In the pre-UIFSM period, there was some evidence of a socioeconomic difference in UPF food intake during school lunchtime (Supplementary Table 4). The highest income group had a higher MPF intake than the lowest income group (48.7%g vs. 41.4%g, respectively) and lower UPF intake (47.8%g vs. 56.4%g). In the DID model, the UIFSM policy was found to have a much greater impact on the lunchtime intake of children from low-income households than children from mid- or high-income households (Fig. 4). For example, low-income children consumed more MPF at lunch after UIFSM when measured by both weight (19.9%g; 95% CI 8.9, 30.9) and energy (20.3%Kcal, 95% CI 11.2,29.4), whereas there was no evidence of a change in mid- or high-income groups. Additionally, the low-income group consumed less UPF at lunch post-UIFSM (-19.3%g, 95% -30.4, − 8.2; -18.3%Kcal, 95% -27.7,-9.0), with no evidence of a change in mid- or high-income children.

### UIFSM policy impact on the quantity of industrial processed food consumed across the school day

The analysis was repeated using the amount of minimally and ultra-processed consumed across the total school day (Supplementary Table 5). The before-after change in the amount of minimally and ultra-processed food consumed across the school day was favourable for the intervention group. However, when the DID model was applied, there was not strong evidence for an impact of the policy across the school day. While the results for minimally processed food (% Kcal Day) reached statistical significance (3.0 MPF % Kcal Day; 95% CI 0.1, 5.9), this was not consistent across all the outcomes. It is likely that the true effect was too small to be detected in this sample.

### Sensitivity analyses

Sensitivity analyses were conducted to assess if the results were robust to different sources of bias. First, participants estimated to be dietary misreporters were excluded and the analyses were repeated (Supplementary Table 6). The confidence intervals were found to overlap, and the point estimates were around 1pp different from the main analyses. Second, participants with one day of dietary data were excluded and the analyses were repeated (Supplementary Table 7). Similarly, the confidence intervals were found to overlap. The point estimates were roughly 2pp different from the main analyses, in the direction of a greater policy impact (main analysis UPF %g -7.5 (CI -13.5,-1.5) vs. sensitivity analysis UPF %g -9.5 (CI -15.8,-3.1)). This therefore suggests that the overall conclusions of the paper were robust to these sources of bias.

## Discussion

### Summary of main findings

This is the first study to evaluate the impact of the UIFSM policy on children’s UPF intake during school lunchtime. We demonstrated that the policy was associated with an overall reduction in UPF intake and an increase in MPF intake during school lunchtime. The effect was driven by a decrease in consuming UPFs associated with packed lunches such as bread, drinks, and salty snacks. Additionally, we observed a socioeconomic gradient in the association, with children from low-income households having greater improvement in both their UPF and MPF consumption at lunch than mid- or high-income children.

### What the study adds to prior knowledge

This study extends the literature evaluating the impact of universal school meal policies on children’s diet and health, by examining the impact on the consumption of ultra-processed foods [20, 32]. As UPFs are associated with children’s health [8], this study further supports the evidence base that indicates universal school meal policies are likely to be beneficial to children’s health [19, 33] and supports the expansion of universal free school meals that is occurring in some areas of the UK [21].

Furthermore, this approach showed that the broadly defined food groups used in previous research, such as ‘Starchy foods’ and ‘Dairy’, may mask important differences by processing level. For example, our research demonstrated a shift from consuming ultra-processed bread to minimally processed starchy foods such as rice and pasta.

### Interpretation of findings and implication for policymakers

We found that school meal uptake has increased substantially following UIFSM, combined with an improvement in the nutritional quality of children’s weekday lunches. Differences in the UPF content between packed lunches and school meals were likely driving the observed effect. There is a ubiquity of packaged convenience foods, which are designed and marketed towards children, including infants, of which a high proportion contain health claims [16, 34, 35]. Indeed, parents report a preference for convenient, packaged, cheap food which they know their child will accept and consume in their packed lunches [36]. This is an issue that affects families of all income levels, as both time and money are quoted as a barrier to preparing minimally processed food for packed lunches, meaning both high and low-income families have reasons for choosing pre-packed and ultra-processed options [36]. Consequently, the UIFSM policy removes a barrier to taking up a school meal for many families and has greater potential to provide healthier, minimally processed options to all children than packed lunches.

However, while our study suggests a positive effect for all children, low-income children benefitted the most from the scheme. Low-income children are the most likely to have a poor quality packed lunch that is higher in UPFs [37], therefore they had the most to gain in switching to a school meal. This is critical as once children are too old to access a Universal Free School Meal, free school meals are provided through a means-tested system in which only the children from very-low-income households are eligible. Indeed, of families receiving social security benefits, only the 30% most deprived are eligible to receive free school meals under the means-tested system [38]. As such, up to one third of children experiencing poverty are not eligible for a means-tested free school meal [39].

There are two factors that could maximise the benefit of the UIFSM policy. First, focussing on increasing school meal uptake. This study estimated the ITT effect, estimating the impact of the UIFSM policy (as opposed to the impact of having school meals) on children’s lunchtime intakes. The ITT effect by construction is smaller, since it is only identified from those who took-up the universally free school meal offered to them. In other words, it is diluted by the 20% of KS1 school children who chose not to take their free school meal. Further encouraging school meal uptake will therefore increase the impact of UIFSM on children’s dietary intakes. Second, improving the quality of food served in school meals. UPFs contributed to around 40% of the weight and 60% of the energy in the post-UIFSM lunches of KS1 schoolchildren. It is also notable that there was no increase in the proportion of children eating minimally processed fruit and vegetables, or a decrease in consuming ultra-processed sweet foods. Working towards increasing the amount of minimally processed food on the menu could increase school meal uptake by alleviating parents’ concerns over the quality of school food [37]. There are examples of voluntary school meal policies in the UK which limit the amount of UPF served. For example, the ‘Food for Life Served Here’ certification goes beyond the statutory School Food Standards and to requires that 75% of the food is freshly prepared [40]. Futhremore, in Brazil a national school meal policy limits the school’s purchase of processed and ultra-processed foods to 20% of the food budget whilst prohibiting certain items such as soft drinks and confectionary [41]. Adopting a similar approach in the national UK School Food Standards or procurement standards could maximise the return on investment in universal free school meals policies [42], through increasing their estimated effect on children’s health and wellbeing. However, schools will need greater support to increase the amount of freshly prepared food served to children. There are many recognised challenges to school food provision in the UK currently [43]. Among these, it is estimated that the funding for free school meals is currently 16% lower in real terms than in 2014 [38]. For the UIFSM to continue having a positive impact, it is essential that schools are given sufficient funding and support to provide healthy food.

### Strengths and limitations

This study is the first to explore the impact of the UIFSM policy on the degree of industrial food processing in children’s food during school lunchtime. This was strengthened using nationally representative data, with detailed dietary information. The quasi-experimental DID method controlled for sources of bias typical in observational studies. This includes accounting for underlying trends which may have affected children’s lunchtime intake. Furthermore, we used more recently collected data than our previous analysis on nutrient outcomes [20]. Finally, the use of IPW weights were used to balance observed characteristics between groups to minimise the impact of selection bias.

There are limitations to note. Sample size issues precluded both the use of multiple time-points and KS1 schoolchildren in Wales and Northern Ireland as the control group, who would have been preferable controls as the intervention was not implemented for KS1 children in these countries (during the study period). While it is not ideal to have children of different ages, the DID design accounted for time-invariant differences between KS1 and KS2 children. Furthermore, there was no other policy or environmental change which would have differentially impacted the lunchtime intake of KS1 children and not KS2 children, to our knowledge. For example, while new school food regulations were introduced in 2015 [44], we do not expect this policy to differentially impact KS1 and KS2 children. Additionally, the intervention and control group are within the same primary school environment. Since our main outcome of interest is UPF intake *at school lunchtime*, any policies that differentially affect KS1 and KS2 children outside school are unlikely to affect our estimates. Evidence comparing the UPF intake of UK children aged 4–7 years and 8–11 years over time is lacking. However, analysis of UPF consumption in US children between 1999 and 2018 shows reasonable evidence of parallel trends in UPF consumption between age groups (2–5 years and 6–11 years) over time [45]. The small sample size may have reduced the power to detect small differences in consumption across the school day. Moreover, it was not possible to exclude children who attended an independent school (where the UIFSM policy does not apply) in the NDNS; however, this is estimated to be around 6% of children, so the impact is likely small [46]. Although it is a strength that 87% of the sample had two or more days of dietary data collected, we note that 13% of the sample only had one day of dietary data, which may not fully capture the variability in school lunchtime intake. However, sensitivity analysis found this did not alter the conclusions of our results (Supplementary Table 7). Furthermore, we recognise that the level of information collected from the NDNS may have led to misclassification of some food items, but this is most likely in a limited number of specific items such as pizza, see Rauber et al. [2] for more details. There was potential bias of dietary misreporting, which was addressed by excluding possible energy misreporters and was found that the results were not substantially altered (Supplementary Table 6).

## Conclusions

This evaluation of the UIFSM policy using natural experiment methods demonstrated that the policy has a positive impact on the diet of schoolchildren at lunchtime in England and Scotland, who ate less ultra-processed food. This policy had a positive impact for all children but demonstrated the most benefit in low-income children. These findings support the expansion of universal free school meal policies and comes at a time when the policy is being extended to wider age ranges across different parts of the United Kingdom, including Wales, Scotland, and London.

## Electronic supplementary material

Below is the link to the electronic supplementary material.

Supplementary Material 1

Supplementary Material 2

## Data Availability

The dataset supporting the conclusions of this article is available in the UK Data Service repository (SN: 6533). 10.5255/UKDA-SN-6533-19.

## Abbreviations

CI: Confidence interval
DID: Difference-in-differences
DINO: Diet in nutrients out
IMD: Index of Multiple Deprivation
IPW: Inverse probability weights
ITT: Intention-to-treat
KS: Key stage
MPF: Minimally processed
NDNS: National Diet and Nutrition Survey
PSU: Primary Sampling Units
SD: Standard deviation
UIFSM: Universal infant free school meal
UK: United Kingdom
UPF: Ultra-processed food
