# Supplementary material for: Evaluating the impact of the universal infant free school meal policy on the ultra-processed food content of children’s lunches in England and Scotland: a natural experiment
Source: Int J Behav Nutr Phys Act. 2024 Nov 1;21:124. doi: 10.1186/s12966-024-01656-w (PMC11528988; doi:10.1186/s12966-024-01656-w)
Supplement: Supplementary file 2 — Supplementary Material 2 [file 12966_2024_1656_MOESM2_ESM.docx]

## Supplementary files

## Title

Evaluating the impact of the Universal Infant Free School Meal policy on the ultra-processed food content of children’s lunches in England and Scotland.

## Authors

Jennie C Parnham PhD, Kiara Chang PhD, Fernanda Rauber PhD, Renata B. Levy PhD, Anthony A Laverty PhD, Jonathan Pearson-Stuttard MD, Martin White MD, Stephanie von Hinke PhD, Christopher Millett PhD, Eszter P Vamos PhD

## List of Tables

[Supplementary Table 1 – Before-and-after difference and DID estimates of the effect of the UIFSM policy on the uptake of school meals in a sample of schoolchildren (n=1,618). 3](#_Toc175219987)

[Supplementary Table 2 – Before-and-after difference and DID estimates of the effect of the UIFSM policy on the proportion of minimally processed foods and ultra-processed foods consumed at lunchtime in a sample of schoolchildren (n=1,618). 4](#_Toc175219988)

[Supplementary Table 3 – Before-after differences and DID estimates of the effect of the UIFSM policy on the proportion of children consuming minimally and ultra-processed food groups at lunchtime in a sample of schoolchildren (n=1,618). 5](#_Toc175219989)

[Supplementary Table 4 – Before-after differences and DID estimates of the effect of the UIFSM policy on the proportion of minimally processed foods and ultra-processed foods consumed at lunchtime in a sample of schoolchildren (n=1,618) and stratified by tertiles of household income. 6](#_Toc175219990)

[Supplementary Table 5 – Before-and-after differences and DID estimates of the effect of the UIFSM policy on the proportion of minimally processed foods and ultra-processed foods consumed throughout the school day in a sample of schoolchildren (n=1,618). 7](#_Toc175219991)

[Supplementary Table 6 - DID estimates of the effect of the UIFSM policy on the proportion of minimally processed foods and ultra-processed foods consumed at school lunch in the main analysis (n=1,618) and a sensitivity analysis with only reliable energy reporters (n=1,552). 8](#_Toc175219992)

[Supplementary Table 7 - DID estimates of the effect of the UIFSM policy on the proportion of minimally processed foods and ultra-processed foods consumed at school lunch in the main analysis (n=1,618) and a sensitivity analysis with only participants who had multiple days of dietary data (n=1,552). 8](#_Toc175219993)

[Supplementary Table 8 -Description of the subsidiary groups within the NOVA food classification system. 9](#_Toc175219994)

Supplementary Table 1 – Before-and-after difference and DID estimates of the effect of the UIFSM policy on the uptake of school meals in a sample of schoolchildren (n=1,618).

| **School meal uptake (%)** | **Pre-UIFSM** | **Post-UIFSM** | **Difference** | **P^1^** | **Crude DiD^2^** | **P** | **Adjusted DiD^3^** | **P** |
| --- | --- | --- | --- | --- | --- | --- | --- | --- |
|  | % | % | Diff. (95% CI) |  | Coef. (95% CI) |  | Coef. (95% CI) |  |
| Intervention | 44.4 | 76.8 | 32.3 (24.7,40.0) | <0.001 | 24.7 (13.5,35.8) | <0.001 | 24.9 (14.0,35.7) | <0.001 |
| Control | 41.6 | 49.3 | 7.7 (-0.3,15.7) | 0.06 |  |  |  |  |
| ^1^Survey weighted t-test; ^2^Unadjusted linear regression; ^3^Linear regression adjusted for age, sex, ethnicity, country, household income and IMD | | | | | | | | |
| SD - standard deviation; CI - confidence interval; IMD – index of multiple deprivation | | | | | | | | |

Supplementary Table 2 – Before-and-after difference and DID estimates of the effect of the UIFSM policy on the proportion of minimally processed foods and ultra-processed foods consumed at lunchtime in a sample of schoolchildren (n=1,618).

| **Variable** | **Pre-UIFSM** | **Post-UIFSM** | **Difference** | **P^1^** | **Model 1^2^** | **P** | **Model 2^3^** | **P** | **Model 3^4^** |  |
| --- | --- | --- | --- | --- | --- | --- | --- | --- | --- | --- |
|  | Mean (SD) | Mean (SD) | Diff. (95% CI) |  | Coef. (95% CI) |  | Coef. (95% CI) |  | Coef. (95% CI) |  |
| **Minimally processed (% g)** |  |  |  |  |  |  |  |  |  |  |
| Intervention | 45.2 (26.0) | 57.6 (24.2) | 12.4 (8.1,16.6) | <0.001 | 8.4 (2.2,14.6) | 0.01 | 8.1 (2.2,14.1) | 0.01 | 7.3 (1.6,13.0) | 0.01 |
| Control | 45.8 (26.9) | 49.8 (27.3) | 4.0 (-0.5,8.5) | 0.08 |  |  |  |  |  |  |
| **UPF (% g)** |  |  |  |  |  |  |  |  |  |  |
| Intervention | 51.6 (26.2) | 40.0 (24.4) | -11.7(-16.0,-7.3) | <0.001 | -7.7 (-14.0,-1.4) | 0.02 | -7.5 (-13.5,-1.5) | 0.01 | -6.8 (-12.5,-1.0) | 0.02 |
| Control | 50.9 (27.1) | 47.0 (27.2) | -3.9 (-8.5,0.6) | 0.09 |  |  |  |  |  |  |
| **Minimally processed (% Kcal)** |  |  |  |  |  |  |  |  |  |  |
| Intervention | 25.2 (21.6) | 33.5 (23.0) | 8.4 (4.6,12.1) | <0.001 | 9.3 (4.3,14.3) | <0.001 | 9.4 (4.5,14.2) | <0.001 | 9.7 (5.0,14.4) | <0.001 |
| Control | 23.7 (21.9) | 22.8 (20.2) | -0.9 (-4.3,2.4) | 0.58 |  |  |  |  |  |  |
| **UPF (% Kcal)** |  |  |  |  |  |  |  |  |  |  |
| Intervention | 67.3 (22.7) | 59.9 (22.7) | -7.4 (-11.2,-3.5) | <0.001 | -6.5 (-11.7,-1.2) | 0.02 | -6.5 (-11.5,-1.5) | 0.01 | -6.8 (-11.7,-1.8) | 0.01 |
| Control | 69.2 (23.0) | 68.2 (21.6) | -0.9 (-4.5,2.6) | 0.60 |  |  |  |  |  |  |
| ^1^Survey weighted t-test; ^2^Model 1 = Unadjusted linear regression; ^3^Model 2 = Linear regression adjusted for age, sex, ethnicity, country, household income and IMD; ^4^Linear regression adjusted for age, sex, ethnicity, country, household income, IMD and total lunchtime intake (g) [for %g variables] or total lunchtime energy (Kcal) [for %Kcal variables] | | | | | | | | | | |
| SD - standard deviation; CI - confidence interval; %g – Percent of total lunchtime grams; % kcal – Percent of total lunchtime calories; IMD – index of multiple deprivation | | | | | | | | | | |

Supplementary Table 3 – Before-after differences and DID estimates of the effect of the UIFSM policy on the proportion of children consuming minimally and ultra-processed food groups at lunchtime in a sample of schoolchildren (n=1,618).

| **Variable** | **Pre-UIFSM** | **Post-UIFSM** | **Difference** | **P^1^** | **Crude DiD^2^** | **P** | **Adjusted DiD^3^** | **P** |
| --- | --- | --- | --- | --- | --- | --- | --- | --- |
|  | % taking | % taking | Diff. (95% CI) |  | Coef. (95% CI) |  | Coef. (95% CI) |  |
| **Minimally processed** |  |  |  |  |  |  |  |  |
| **Drinks** |  |  |  |  |  |  |  |  |
| Intervention | 61.6 | 72.7 | 11.1 (3.3,18.9) | 0.01 | 2.5 (-8.4,13.4) | 0.65 | 1.8 (-8.6,12.2) | 0.73 |
| Control | 62.4 | 70.9 | 8.5 (0.9,16.2) | 0.03 |  |  |  |  |
| **Fruit & veg** |  |  |  |  |  |  |  |  |
| Intervention | 80.6 | 81.6 | 1.0 (-5.8,7.7) | 0.78 | -2.3 (-12.4,7.7) | 0.65 | -2.4 (-12.2,7.5) | 0.64 |
| Control | 72.2 | 75.5 | 3.3 (-4.2,10.7) | 0.39 |  |  |  |  |
| **Dairy & eggs** |  |  |  |  |  |  |  |  |
| Intervention | 13.3 | 23.6 | 10.3 (3.1,17.4) | <0.01 | 11.4 (2.8,20.0) | 0.01 | 11.4 (3.1,19.6) | 0.01 |
| Control | 11.7 | 10.6 | -1.1 (-5.9,3.7) | 0.64 |  |  |  |  |
| **Starchy foods & legumes** |  |  |  |  |  |  |  |  |
| Intervention | 37.6 | 60.4 | 22.8 (14.3,31.3) | <0.01 | 19.1 (7.6,30.7) | <0.01 | 19.2 (8.2,30.3) | <0.01 |
| Control | 36.5 | 40.1 | 3.6 (-4.1,11.4) | 0.36 |  |  |  |  |
| **Meat & fish** |  |  |  |  |  |  |  |  |
| Intervention | 31.3 | 43.9 | 12.6 (4.0,21.2) | <0.01 | 12.4 (1.0,23.8) | 0.03 | 12.3 (1.2,23.4) | 0.03 |
| Control | 33.0 | 33.2 | 0.2 (-7.3,7.7) | 0.96 |  |  |  |  |
| **Ultra-processed** |  |  |  |  |  |  |  |  |
| **UP bread** |  |  |  |  |  |  |  |  |
| Intervention | 69.5 | 52.1 | -17.4 (-26.0,-8.9) | <0.01 | -15.1 (-26.4,-3.8) | 0.01 | -14.9 (-25.9,-4.0) | 0.01 |
| Control | 69.5 | 67.2 | -2.3 (-9.8,5.1) | 0.54 |  |  |  |  |
| **Salty snacks** |  |  |  |  |  |  |  |  |
| Intervention | 22.6 | 13.8 | -8.8 (-14.6,-3.0) | <0.01 | -13.0 (-22.4,-3.7) | 0.01 | -12.6 (-21.8,-3.5) | 0.01 |
| Control | 26.5 | 30.8 | 4.3 (-3.1,11.6) | 0.25 |  |  |  |  |
| **UP drinks** |  |  |  |  |  |  |  |  |
| Intervention | 41.2 | 27.1 | -14.1 (-23.1,-5.1) | <0.01 | -12.3 (-24.2,-0.3) | 0.04 | -12.5 (-23.6,-1.4) | 0.03 |
| Control | 36.4 | 34.5 | -1.8 (-9.7,6.0) | 0.64 |  |  |  |  |
| **Sweet foods** |  |  |  |  |  |  |  |  |
| Intervention | 64.0 | 73.2 | 9.2 (1.4,16.9) | 0.02 | 4.8 (-5.7,15.2) | 0.37 | 4.9 (-5.3,15.0) | 0.34 |
| Control | 73.4 | 77.8 | 4.4 (-2.6,11.4) | 0.22 |  |  |  |  |
| **Ready-to-eat** |  |  |  |  |  |  |  |  |
| Intervention | 82.8 | 86.0 | 3.1 (-2.3,8.6) | 0.26 | 5.5 (-3.0,14.0) | 0.21 | 5.4 (-3.2,13.9) | 0.22 |
| Control | 82.0 | 79.7 | -2.3 (-8.8,4.2) | 0.48 |  |  |  |  |
| **UP Dairy** |  |  |  |  |  |  |  |  |
| Intervention | 47.1 | 27.5 | -19.6 (-27.3,-11.9) | <0.01 | -9.3 (-20.1,1.6) | 0.10 | -8.9 (-19.6,1.9) | 0.11 |
| Control | 45.0 | 34.6 | -10.4 (-18.1,-2.6) | 0.01 |  |  |  |  |
| **UP Meat & Fish** |  |  |  |  |  |  |  |  |
| Intervention | 49.1 | 45.9 | -3.2 (-12.0,5.5) | 0.47 | -4.8 (-16.7,7.0) | 0.42 | -4.7 (-16.2,6.7) | 0.42 |
| Control | 47.2 | 48.8 | 1.6 (-6.4,9.7) | 0.69 |  |  |  |  |
| **UP Veg** |  |  |  |  |  |  |  |  |
| Intervention | 13.7 | 23.1 | 9.4 (1.7,17.1) | 0.02 | 5.6 (-3.6,14.8) | 0.24 | 5.4 (-3.3,14.0) | 0.23 |
| Control | 10.1 | 13.9 | 3.8 (-1.3,8.9) | 0.15 |  |  |  |  |
| ^1^Survey weighted t-test; ^2^Unadjusted linear probability regression; ^3^Linear probability regression adjusted for age, sex, ethnicity, household size, region, household income and IMD | | | | | | | | |
| SD - standard deviation; CI - confidence interval; IMD – index of multiple deprivation | | | | | | | | |

Supplementary Table 4 – Before-after differences and DID estimates of the effect of the UIFSM policy on the proportion of minimally processed foods and ultra-processed foods consumed at lunchtime in a sample of schoolchildren (n=1,618) and stratified by tertiles of household income.

| **Variable** | **Low-income Pre-UIFSM** | **Difference** | **Mid-income Pre-UIFSM** | **Difference** | **High-income Pre-UIFSM** | **Difference** | **Low-income DiD^2^** | **P** | **Mid-income DiD^2^** | **P** | **High-Income DiD^2^** | **P** |
| --- | --- | --- | --- | --- | --- | --- | --- | --- | --- | --- | --- | --- |
|  | Mean (SD) | Diff. (95% CI)^1^ | Mean (SD) | Diff. (95% CI)^1^ | Mean (SD) | Diff. (95% CI) | Coef. (95% CI) |  | Coef. (95% CI) |  | Coef. (95% CI) |  |
| **Minimally processed (% g)** |  |  |  |  |  |  |  |  |  |  |  |  |
| Intervention | 41.4 (26.6) | 16.6* (8.0,25.2) | 45.5 (26.7) | 9.0* (2.6,15.5) | 48.7 (24.3) | 11.7* (4.5,18.9) | 19.9 (8.9,30.9) | <0.01 | 5.1 (-4.4,14.5) | 0.29 | 1.6 (-8.0,11.2) | 0.75 |
| Control | 44.1 (28.2) | -1.3 (-9.8,7.2) | 46.3 (27.7) | 3.0 (-4.3,10.2) | 47.0 (24.5) | 10.9* (3.8,18.0) |  |  |  |  |  |  |
| **Minimally processed (% kcal)** |  |  |  |  |  |  |  |  |  |  |  |  |
| Intervention | 24.4 (22.3) | 10.5* (3.2,17.8) | 25.1 (21.9) | 3.8 (-2.0,9.6) | 26.0 (20.7) | 10.9* (4.6,17.2) | 20.3 (11.2,29.4) | <0.01 | 5.1 (-2.3,12.4) | 0.17 | 5.6 (-2.5,13.7) | 0.18 |
| Control | 26.8 (24.4) | -7.4* (-13.7,-1.1) | 22.8 (21.8) | -1.5 (-6.5,3.6) | 21.8 (18.9) | 6.6* (0.7,12.4) |  |  |  |  |  |  |
| **UPF (% g)** |  |  |  |  |  |  |  |  |  |  |  |  |
| Intervention | 56.4 (26.8) | -16.3* (-25.1,-7.5) | 50.8 (26.9) | -7.8* (-14.5,-1.1) | 47.8 (24.2) | -11.1* (-18.4,-3.9) | -19.3 (-30.4,-8.2) | <0.01 | -3.8 (-13.4,5.9) | 0.44 | -1.8 (-11.4,7.7) | 0.70 |
| Control | 53.2 (27.7) | 1.0 (-7.7,9.7) | 50.3 (28.1) | -3.0 (-10.2,4.3) | 49.3 (25.1) | -10.2* (-17.2,-3.2) |  |  |  |  |  |  |
| **UPF (% kcal)** |  |  |  |  |  |  |  |  |  |  |  |  |
| Intervention | 69.8 (23.1) | -10.9* (-18.8,-3.0) | 66.1 (23.6) | -1.7 (-7.5,4.1) | 66.2 (20.9) | -9.9* (-16.2,-3.7) | -18.3 (-27.7,-9.0) | <0.01 | 0.3 (-7.5,8.1) | 0.94 | -5.3 (-13.5,3.0) | 0.21 |
| Control | 67.5 (24.4) | 4.8 (-2.1,11.7) | 69.9 (23.5) | -1.7 (-7.2,3.8) | 70.0 (20.8) | -5.9 (-12.1,0.3) |  |  |  |  |  |  |
| ^1^Survey weighted t-test for difference between pre- and post-UIFSM values ^2^Linear regression adjusted for sex, ethnicity and IMD | | | | | | | | | | | | |
| * P<0.05; SD - standard deviation; CI - confidence interval; MPF – Minimally processed food; %g – Percent of total lunchtime grams; % kcal – Percent of total lunchtime calories; UPF – Ultra-processed food; IMD – index of multiple deprivation | | | | | | | | | | | | |

Supplementary Table 5 – Before-and-after differences and DID estimates of the effect of the UIFSM policy on the proportion of minimally processed foods and ultra-processed foods consumed throughout the school day in a sample of schoolchildren (n=1,618).

| **Variable** | **Pre-UIFSM** | **Post-UIFSM** | **Difference** | **P^1^** | **Crude DiD^2^** | **P** | **Adjusted DiD^3^** | **P** |
| --- | --- | --- | --- | --- | --- | --- | --- | --- |
|  | Mean (SD) | Mean (SD) | Diff. (95% CI) |  | Coef. (95% CI) |  | Coef. (95% CI) |  |
| **Minimally processed (% g day)** |  |  |  |  |  |  |  |  |
| Intervention | 55.3 (17.0) | 59.8 (19.4) | 4.5 (1.5,7.4) | <0.001 | 1.9 (-2.7,6.5) | 0.42 | 1.5 (-2.6,5.7) | 0.47 |
| Control | 54.3 (18.2) | 56.8 (21.3) | 2.6 (-1.0,6.1) | 0.16 |  |  |  |  |
| **Minimally processed (% kcal day)** |  |  |  |  |  |  |  |  |
| Intervention | 30.2 (12.0) | 31.0 (13.8) | 0.8 (-1.4,2.9) | 0.48 | 3.0 (-0.0,6.1) | 0.05 | 3.0 (0.1,5.9) | 0.04 |
| Control | 28.0 (13.4) | 25.8 (12.7) | -2.2 (-4.4,-0.0) | 0.05 |  |  |  |  |
| **UPF (% g day)** |  |  |  |  |  |  |  |  |
| Intervention | 42.5 (17.2) | 38.5 (19.6) | -4.0 (-7.0,-1.0) | 0.01 | -1.6 (-6.3,3.1) | 0.51 | -1.2 (-5.5,3.0) | 0.56 |
| Control | 43.8 (18.3) | 41.4 (21.5) | -2.4 (-6.0,1.2) | 0.20 |  |  |  |  |
| **UPF (% kcal day)** |  |  |  |  |  |  |  |  |
| Intervention | 63.3 (13.9) | 62.5 (14.9) | -0.8 (-3.2,1.6) | 0.51 | -2.3 (-5.8,1.2) | 0.19 | -2.2 (-5.5,1.0) | 0.18 |
| Control | 66.0 (14.4) | 67.5 (14.5) | 1.5 (-1.0,4.0) | 0.24 |  |  |  |  |
| ^1^Survey adjusted t-test; ^2^Unadjusted linear regression; ^3^Linear regression adjusted for age, sex, ethnicity, country, household income, IMD, total energy and total lunchtime intake (g); UPF = ultra-processed food. | | | | | | | | |
| SD - standard deviation; CI - confidence interval; %g – Percent of total lunchtime grams; % kcal – Percent of total lunchtime calories; IMD – index of multiple deprivation | | | | | | | | |

Supplementary Table 6 - DID estimates of the effect of the UIFSM policy on the proportion of minimally processed foods and ultra-processed foods consumed at school lunch in the main analysis (n=1,618) and a sensitivity analysis with only reliable energy reporters (n=1,552).

| **Variable** | **Main analysis** (n=1618) Unadjusted | P | **Main analysis** (n=1618) Adjusted | P | **Reliable reporters** (n=1552) Unadjusted | P | **Reliable reporters** (n=1552) Adjusted | P |
| --- | --- | --- | --- | --- | --- | --- | --- | --- |
| **Minimally processed (% g)** | 8.4 (2.2,14.6) | 0.01 | 8.1 (2.2,14.1) | 0.01 | 7.6 (1.3,13.9) | 0.02 | 7.5 (1.4,13.6) | 0.02 |
| **Minimally processed (% kcal)** | 9.3 (4.3,14.3) | <0.01 | 9.4 (4.5,14.2) | <0.01 | 8.6 (3.5,13.6) | <0.01 | 8.7 (3.8,13.6) | <0.01 |
| **UPF (% g)** | -7.7 (-14.0,-1.4) | 0.02 | -7.5 (-13.5,-1.5) | 0.01 | -6.9 (-13.3,-0.5) | 0.03 | -6.8 (-12.9,-0.7) | 0.03 |
| **UPF (% kcal)** | -6.5 (-11.7,-1.2) | 0.02 | -6.5 (-11.5,-1.5) | 0.01 | -5.6 (-11.0,-0.3) | 0.04 | -5.8 (-11.0,-0.7) | 0.03 |
| Note: Main analysis includes all participants. Reliable energy reporters includes only participants whose reported energy was within their estimated energy requirement (Under-[n=64] and over-reporters [n=2] removed); Unadjusted - Linear regression not adjusted for covariates; Adjusted - Linear regression adjusted for age, sex, ethnicity, country, household income and IMD; %g – Percent of total lunchtime grams; % kcal – Percent of total lunchtime calories; IMD – index of multiple deprivation | | | | | | | | |

Supplementary Table 7 - DID estimates of the effect of the UIFSM policy on the proportion of minimally processed foods and ultra-processed foods consumed at school lunch in the main analysis (n=1,618) and a sensitivity analysis with only participants who had multiple days of dietary data (n=1,552).

| **Variable** | **Main analysis** (n=1618) Unadjusted | P | **Main analysis** (n=1618) Adjusted | P | **Multiple intakes only** (n=1411) Unadjusted | P | **Multiple intakes only** (n=1411) Adjusted | P |
| --- | --- | --- | --- | --- | --- | --- | --- | --- |
| **Minimally processed (% g)** | 8.4 (2.2,14.6) | 0.01 | 8.1 (2.2,14.1) | 0.01 | 10.2 (3.7,16.8) | <0.01 | 10.2 (3.9,16.5) | <0.01 |
| **Minimally processed (% kcal)** | 9.3 (4.3,14.3) | <0.01 | 9.4 (4.5,14.2) | <0.01 | 9.8 (4.6,15.0) | <0.01 | 10.0 (5.0,15.1) | <0.01 |
| **UPF (% g)** | -7.7 (-14.0,-1.4) | 0.02 | -7.5 (-13.5,-1.5) | 0.01 | -9.4 (-16.1,-2.8) | 0.01 | -9.5 (-15.8,-3.1) | <0.01 |
| **UPF (% kcal)** | -6.5 (-11.7,-1.2) | 0.02 | -6.5 (-11.5,-1.5) | 0.01 | -7.3 (-12.8,-1.8) | 0.01 | -7.5 (-12.8,-2.3) | 0.01 |
| **Note**: Main analysis includes all participants (n=1618). 'Multiple intakes only' is a sensitivity analysis which only included participants with two or more recorded school lunchtime intakes (participants with one intake (n=207) were excluded); Unadjusted - Linear regression not adjusted for covariates; Adjusted - Linear regression adjusted for age, sex, ethnicity, country, household income, IMD and total lunch (g); %g – Percent of total lunchtime grams; % kcal – Percent of total lunchtime calories; IMD – index of multiple deprivation | | | | | | | | |

Supplementary Table 8 -Description of the subsidiary groups within the NOVA food classification system.

| **Subsidary Group** | **Definition** |
| --- | --- |
| **NOVA 1—minimally processed food (MPF)** |  |
| Drinks | Water, coffee and tea, fresh fruit juices and smoothies |
| Fruit and vegetables | Fruit, vegetables, fungi, nuts and seeds |
| Dairy and eggs | Milk, plain yoghurt, eggs |
| Starchy foods and legumes | Grains, legumes, pasta, homemade pies and pastries |
| Meat and fish | Fish, poultry, red meat, pies and pastries with meat or fish, seafood |
| **NOVA 4—Ultra-processed (UPF)** |  |
| Processed bread | Industrially manufactured bread |
| Sweet foods | Industrially manufactured cakes, pies, biscuits, sweet snacks, ice cream, ice pops, desserts, sweet spreads and icing, artificial sugars and sweeteners |
| Salty snacks | Crisps and other salty snacks |
| Drinks | Soft drinks (high and low calorie) and fruit drinks |
| Ready-to-eat foods | Pasta and rice dishes (ready-to-eat/heat), egg and cheese dishes (ready-to-eat/heat), bacon/sausages dishes (ready-to-eat/heat), meat dishes (ready-to-eat/heat)  Chicken/turkey dishes (ready-to-eat/heat), fish dishes (ready-to-eat/heat), vegetables dishes (ready-to-eat/heat), meat alternatives  Potato dishes (ready-to-eat/heat), instant and canned soups, industrially manufactured meat pies and pastries  Pizza, French fries and other potato products, sandwiches and hamburgers  Sauces, dressings, gravy, spread, margarine |
| Ultra-processed dairy, meat, and fish | Industrially processed meat and fish (inc. bacon, ham), Industrially manufactured yoghurts and milk drinks, Processed cheese and cheese products |
| Vegetables | Industrially processed vegetables (baked beans, processed peas) |
